# Supplementary material for: NK cell exhaustion in Wilson’s disease revealed by single-cell RNA sequencing predicts the prognosis of cholecystitis
Source: eLife. 2024 Dec 30;13:RP98867. doi: 10.7554/eLife.98867 (PMC11684787; doi:10.7554/eLife.98867)
Supplement: Supplementary file 1. — (a) The number and proportion of each cell type. This table shows the number and proportion of each cell type and each subtype in all samples. (b) The list of genes in each cluster. This table shows the gene ID included in three clusters, and the genes mentioned in the article are marked in yellow. (c) The list of genes used for signature scoring. This table shows the gene ID used for signature scoring. (d) The list of marker genes used for cell type annotation. This table shows the marker genes used for cell type and subtype annotation and the respective references. [file elife-98867-supp1.pdf]

Supplementary File 1a

The number of each celltype	cluster	CASE_1	CASE_2	CASE_3	Sum	CON_1	CON_2	CON_3	Sum
Main	Hepatocytes	173	318	133	624	170	132	27	329
	Cholangiocytes	74	59	714	847	21	83	53	157
	ECs	1060	303	1155	2518	187	574	237	998
	HepSCs	71	32	213	316	22	239	88	349
	ProliferatingCells	187	30	96	313	16	23	37	76
	BCells	467	370	498	1335	304	158	145	607
	PlasmaCells	32	10	687	729	33	74	8	115
	TandNK	6255	5137	5130	16522	6915	5199	7915	20029
	Neutrophils	1175	35	63	1273	95	1407	304	1806
	MastCells	17	10	41	68	17	22	26	65
	MPs	2774	1122	1488	5384	641	1434	666	2741
	pDCs	76	12	51	139	11	12	21	44
	Sum	12361	7438	10269	30068	8432	9357	9527	27316
MPs	ProliferatingCells	18	7	6	31	0	2	1	3
	Macrophages	566	235	300	1101	87	179	84	350
	Monocytes	505	595	287	1387	140	889	211	1240
	MatureDCs	5	1	6	12	2	8	7	17
	cDC1	62	35	4	101	52	65	22	139
	cDC2	265	79	130	474	68	75	147	290
	KCs	922	75	336	1333	192	161	131	484
	Sum	2343	1027	1069	4439	541	1379	603	2523
TandNK	ILC3_IL1R1	21	10	33	64	9	17	9	35
	NKT_GNLY	979	399	270	1648	725	674	831	2230
	NKT_IFNG	288	524	132	944	639	201	446	1286
	NKT_KLRG1	168	483	204	855	950	245	591	1786
	NK_FCER1G	1914	859	1729	4502	210	887	369	1466
	NK_KLRC1	280	90	485	855	54	157	64	275
	NKT_XCL2	206	661	147	1014	799	205	465	1469
	NK_FCGR3A	474	230	230	934	238	579	344	1161
	CD4NaiveT_CCR7	197	192	109	498	248	260	128	636
	CD4Tmem_CD40LG	201	105	163	469	406	111	159	676
	CD4Treg_CTLA4	45	13	78	136	25	15	19	59
	CD8MAIT_KLRB1	263	273	169	705	356	430	1199	1985
	CD8MAIT_SLC4A10	254	175	80	509	324	453	1651	2428
	CD8Teff_GZMH	408	624	381	1413	835	294	554	1683
	CD8Teff_GZMK	221	200	247	668	669	357	659	1685
	CD8Teff_ISG15	6	0	1	7	1	2	1	4
	CD8Teff_MT1X	74	79	29	182	53	7	12	72
	Sum	5999	4917	4487	15403	6541	4894	7501	18936

The proportion of each celltype %	cluster	CASE_1	CASE_2	CASE_3	Average	CON_1	CON_2	CON_3	Average
Main	Hepatocytes	1.400	4.275	1.295	2.323	2.016	1.411	0.283	1.237
	Cholangiocytes	0.599	0.793	6.953	2.782	0.249	0.887	0.556	0.564
	ECs	8.575	4.074	11.247	7.965	2.218	6.134	2.488	3.613
	HepSCs	0.574	0.430	2.074	1.026	0.261	2.554	0.924	1.246
	ProliferatingCells	1.513	0.403	0.935	0.950	0.190	0.246	0.388	0.275
	BCells	3.778	4.974	4.850	4.534	3.605	1.689	1.522	2.272
	PlasmaCells	0.259	0.134	6.690	2.361	0.391	0.791	0.084	0.422
	TandNK	50.603	69.064	49.956	56.541	82.009	55.563	83.080	73.550
	Neutrophils	9.506	0.471	0.613	3.530	1.127	15.037	3.191	6.451
	MastCells	0.138	0.134	0.399	0.224	0.202	0.235	0.273	0.237
	MPs	22.442	15.085	14.490	17.339	7.602	15.325	6.991	9.973
	pDCs	0.615	0.161	0.497	0.424	0.130	0.128	0.220	0.160
MPs	ProliferatingCells	0.768	0.682	0.561	0.670	0.000	0.145	0.166	0.104
	Macrophages	24.157	22.882	28.064	25.034	16.081	12.980	13.930	14.331
	Monocytes	21.554	57.936	26.848	35.446	25.878	64.467	34.992	41.779
	MatureDCs	0.213	0.097	0.561	0.291	0.370	0.580	1.161	0.704
	cDC1	2.646	3.408	0.374	2.143	9.612	4.714	3.648	5.991
	cDC2	11.310	7.692	12.161	10.388	12.569	5.439	24.378	14.129
	KCs	39.351	7.303	31.431	26.028	35.490	11.675	21.725	22.963
	ILC3_IL1R1	0.350	0.203	0.735	0.430	0.138	0.347	0.120	0.202
	NKT_GNLY	16.319	8.115	6.017	10.150	11.084	13.772	11.079	11.978
	NKT_IFNG	4.801	10.657	2.942	6.133	9.769	4.107	5.946	6.607
	NKT_KLRG1	2.800	9.823	4.546	5.723	14.524	5.006	7.879	9.136
	NK_FCER1G	31.905	17.470	38.534	29.303	3.211	18.124	4.919	8.751
	NK_KLRC1	4.667	1.830	10.809	5.769	0.826	3.208	0.853	1.629
	NKT_XCL2	3.434	13.443	3.276	6.718	12.215	4.189	6.199	7.534

	NK_FCGR3A	7.901	4.678	5.126	5.902	3.639	11.831	4.586	6.685
TandNK	CD4NaiveT_CCR7	3.284	3.905	2.429	3.206	3.791	5.313	1.706	3.604
	CD4Tmem_CD40LG	3.351	2.135	3.633	3.040	6.207	2.268	2.120	3.532
	CD4Treg_CTLA4	0.750	0.264	1.738	0.918	0.382	0.306	0.253	0.314
	CD8MAIT_KLRB1	4.384	5.552	3.766	4.568	5.443	8.786	15.985	10.071
	CD8MAIT_SLC4A10	4.234	3.559	1.783	3.192	4.953	9.256	22.010	12.073
	CD8Teff_GZMH	6.801	12.691	8.491	9.328	12.766	6.007	7.386	8.720
	CD8Teff_GZMK	3.684	4.068	5.505	4.419	10.228	7.295	8.785	8.769
	CD8Teff_ISG15	0.100	0.000	0.022	0.041	0.015	0.041	0.013	0.023
	CD8Teff_MT1X	1.234	1.607	0.646	1.162	0.810	0.143	0.160	0.371

Supplementary File 1b

	gene_id	Cluster
1	ACTB	1
2	RPS26	1
3	GIMAP7	1
4	IGKC	1
5	FCER1G	1
6	GSTP1	1
7	ACTG1	1
8	CCL5	1
9	AC007952.4	1
10	KLRD1	1
11	CLIC3	1
12	CD7	1
13	TMSB4X	1
14	GZMA	1
15	RSRP1	1
16	GAPDH	1
17	MYL12A	1
18	KLRC1	1
19	CD247	1
20	ID2	1
21	XIST	1
22	PFN1	1
23	CLIC1	1
24	SOCS1	1
25	GZMK	1
26	CORO1A	1
27	HCST	1
28	HLA-B	1
29	TXK	1
30	CITED2	1
31	ATP5F1E	1
32	TYROBP	1
33	IFITM2	1
34	LY6E	1
35	SESN1	1
36	LSP1	1
37	CD63	1
38	CTSD	1
39	EVL	1
40	PSME1	1
41	FKBP5	1
42	CFL1	1
43	TMIGD2	1
44	GADD45G	1
45	GABPB1-AS	1
46	RAC2	1
47	ARPC3	1
48	GADD45B	1
49	ARRDC3	1
50	CD160	1
51	ETS1	1
52	MYL12B	1
53	PTGDR	1
54	AC104506.1	1
55	ZBTB16	1
56	MAPK1	1
57	IGHA1	1
58	RARRES3	1
59	INTS6	1
60	FUS	1
61	ILF3-DT	1
62	TRAPPC1	1
63	GZMM	1
64	RASA2	1
65	EIF3G	1
66	AL355075.4	1

	gene_id	Cluster
757	ARMC6	1
758	DHPS	1
759	ELP3	1
760	MRI1	1
761	CD38	1
762	AC022021.1	1
763	PNKD	1
764	C12orf10	1
765	FBXL6	1
766	CLUAP1	1
767	OFD1	1
768	COX5A	1
769	ANKRD10	1
770	ATP5S	1
771	TBC1D10A	1
772	METTL26	1
773	TARSL2	1
774	TAF9	1
775	ADD1	1
776	DEXI	1
777	SYNJ2BP	1
778	TMEM106C	1
779	TMOD3	1
780	CLNS1A	1
781	STUB1	1
782	DPM3	1
783	ATP6V1F	1
784	ZNF567	1
785	ECHS1	1
786	NAGLU	1
787	MAF1	1
788	ATP5F1C	1
789	LBR	1
790	TEFM	1
791	APH1A	1
792	POLR2I	1
793	CAMK1	1
794	RTRAF	1
795	BTF3L4	1
796	ZFP14	1
797	METTL23	1
798	FAM50B	1
799	COMMD9	1
800	CSK	1
801	SDAD1	1
802	TINF2	1
803	IMP3	1
804	SF3B5	1
805	APEX1	1
806	ZNF428	1
807	METTL3	1
808	CFAP97	1
809	TERF2IP	1
810	STX10	1
811	RALY	1
812	TRAPPC2L	1
813	CENPT	1
814	SLC7A6OS	1
815	C11orf58	1
816	ZNF75A	1
817	TUBB	1
818	VPS26A	1
819	BCL2	1
820	PDCD6	1
821	SP110	1
822	ITGA4	1

	gene_id	Cluster
1517	EGR2	2
1518	BTG3	2
1519	HIC1	2
1520	GZF1	2
1521	PTGES3	2
1522	HMGCS1	2
1523	ARG2	2
1524	SLC25A33	2
1525	CNOT2	2
1526	UBE2B	2
1527	AHSA1	2
1528	OAZ1	2
1529	HNRNPC	2
1530	AMD1	2
1531	CHD4	2
1532	MIDN	2
1533	SERP1	2
1534	NAA50	2
1535	MOB3A	2
1536	KBTBD2	2
1537	YME1L1	2
1538	SLC2A3	2
1539	MAT2A	2
1540	AC103591.3	2
1541	BIRC2	2
1542	TNFSF14	2
1543	POLR2K	2
1544	EHD1	2
1545	CLK1	2
1546	KPNA2	2
1547	PABPC4	2
1548	SUB1	2
1549	CCT4	2
1550	TSC22D2	2
1551	RPLP1	2
1552	MIR222HG	2
1553	SFPQ	2
1554	RPL35	2
1555	TIFA	2
1556	SKP1	2
1557	TMEM263	2
1558	NBEAL1	2
1559	UBE2A	2
1560	ZBTB21	2
1561	LEPROTL1	2
1562	ZBTB10	2
1563	FAM107B	2
1564	TAF13	2
1565	GOLGB1	2
1566	SERTAD3	2
1567	SLC1A5	2
1568	RFX1	2
1569	AZIN1	2
1570	ZDBF2	2
1571	NSMCE3	2
1572	MKNK2	2
1573	BCL2L11	2
1574	NFKB2	2
1575	H2AFJ	2
1576	NFKBIE	2
1577	UBE2D3	2
1578	RHOH	2
1579	THUMPD3-	2
1580	IFITM1	2
1581	TOB2	2
1582	EGR3	2

67	SERF2	1	823	MLEC	1	1583	NASP	2
68	GIMAP4	1	824	ASAH1	1	1584	SIRT1	2
69	ITM2C	1	825	TMEM203	1	1585	NAMPT	2
70	TMA7	1	826	C8orf59	1	1586	RIPK2	2
71	TRDC	1	827	DDIT4	1	1587	ICAM4	2
72	C4orf3	1	828	TMEM42	1	1588	ALG13	2
73	CXCR6	1	829	CLNK	1	1589	THAP2	2
74	NDUFA4	1	830	PGBD4	1	1590	GCH1	2
75	TLN1	1	831	SPCS1	1	1591	IFNGR1	2
76	GIMAP1	1	832	CDC16	1	1592	HIST2H2AC	2
77	PSME2	1	833	TSPAN5	1	1593	PDE7A	2
78	UQCR11	1	834	TRMT10B	1	1594	SRSF7	2
79	PPIB	1	835	AUTS2	1	1595	PNPLA8	2
80	ELOB	1	836	TBRG4	1	1596	MMP9	2
81	GGNBP2	1	837	PLP2	1	1597	RBM38	2
82	SPCS2	1	838	XRCC5	1	1598	ATG101	2
83	CHST12	1	839	AC243829.1	1	1599	NLRP3	2
84	RHOC	1	840	NIFK	1	1600	CASP3	2
85	PRELID1	1	841	ZNF302	1	1601	JPT1	2
86	ARHGDIB	1	842	ARSK	1	1602	TANK	2
87	AP001160.1	1	843	SNAPC5	1	1603	ISG20L2	2
88	AL627171.1	1	844	GHITM	1	1604	CNOT6L	2
89	RHOA	1	845	COMMD2	1	1605	SIK1B	2
90	ENO1	1	846	COMTD1	1	1606	RHEB	2
91	IKZF1	1	847	WDR1	1	1607	LIF	2
92	AC108134.2	1	848	DNMT3A	1	1608	SC5D	2
93	PPP1CA	1	849	SYK	1	1609	MAP1LC3A	2
94	PPP1R14B	1	850	TMEM126B	1	1610	USP36	2
95	ATP5MC2	1	851	U2AF1L5	1	1611	RAB21	2
96	IGHM	1	852	TMCO1	1	1612	RYBP	2
97	CDC42	1	853	MPC1	1	1613	TFRC	2
98	ACAP1	1	854	CIR1	1	1614	DDX21	2
99	IFI16	1	855	RBL2	1	1615	ELOVL5	2
100	OST4	1	856	CCDC124	1	1616	DBF4	2
101	PNISR	1	857	SFXN1	1	1617	KDM2A	2
102	PLEKHF1	1	858	TSNAX	1	1618	RNF168	2
103	DAD1	1	859	M6PR	1	1619	DUSP6	2
104	PYHIN1	1	860	MRPS5	1	1620	SIK2	2
105	PRDX5	1	861	DCP2	1	1621	DNAJB9	2
106	CAP1	1	862	CCDC85B	1	1622	AKIRIN1	2
107	ALOX5AP	1	863	SH2D1B	1	1623	RARA	2
108	APMAP	1	864	RCBTB2	1	1624	LRIF1	2
109	BST2	1	865	H6PD	1	1625	MAP2K3	2
110	EOMES	1	866	RBM43	1	1626	EIF1AY	2
111	SET	1	867	SUMO2	1	1627	PPP1CB	2
112	LAT2	1	868	OGT	1	1628	CRYBG1	2
113	ARGLU1	1	869	PSMF1	1	1629	RASL11A	2
114	FASLG	1	870	ZNF439	1	1630	MOB4	2
115	CD53	1	871	VDAC1	1	1631	DYNLL2	2
116	TBC1D10C	1	872	PARK7	1	1632	CYSLTR2	2
117	IFIT2	1	873	A1BG	1	1633	CCNT1	2
118	MSN	1	874	EMC1	1	1634	ISCA1	2
119	TMEM141	1	875	CKLF	1	1635	CMC1	2
120	ARL6IP4	1	876	PAK2	1	1636	SRSF5	2
121	EIF5A	1	877	FNTA	1	1637	SNHG8	2
122	HMGB1	1	878	UBE2D2	1	1638	TRA2A	2
123	PSMB8	1	879	PIGBOS1	1	1639	LUZP1	2
124	PTPRC	1	880	NDUFAB1	1	1640	NKRF	2
125	IGHG3	1	881	CHRNB1	1	1641	PPP2CA	2
126	C19orf66	1	882	COX14	1	1642	PIM2	2
127	PIK3IP1	1	883	AL451085.1	1	1643	ISG15	2
128	HMGN1	1	884	CTBP1	1	1644	EGR4	2
129	ANXA6	1	885	BUB3	1	1645	MORF4L2	2
130	ECH1	1	886	NUTF2	1	1646	NDUFS5	2
131	HSD17B10	1	887	HINT2	1	1647	TOPORS	2
132	CSKMT	1	888	MRPL28	1	1648	AL359915.2	2
133	PAXX	1	889	RTP4	1	1649	ZNF571	2
134	ATP5MD	1	890	ENPP4	1	1650	AURKA	2

135	VAMP8	1	891	FARSA	1	1651	GADD45A	2
136	GMFG	1	892	AC017083.1	1	1652	CCDC117	2
137	WDR83OS	1	893	MLH3	1	1653	TRIM39	2
138	GTF3A	1	894	NIFK-AS1	1	1654	TGIF2	2
139	MYL6	1	895	BOD1	1	1655	ATP1A1	2
140	ITGB2	1	896	SASH3	1	1656	IRF2BP2	2
141	TRAF3IP3	1	897	ZDHHC24	1	1657	TMEM217	2
142	PSMB3	1	898	C19orf24	1	1658	EMD	2
143	COX6B1	1	899	CNDP2	1	1659	ZFAS1	2
144	NT5C	1	900	NOL7	1	1660	SLC35A2	2
145	GPATCH8	1	901	CCDC28A	1	1661	CD72	2
146	ARPC4	1	902	ARHGDIA	1	1662	SRSF3	2
147	IGLC2	1	903	ARL6IP5	1	1663	BTBD7	2
148	CAPZB	1	904	IER3IP1	1	1664	RABGEF1	2
149	MDH2	1	905	IFI35	1	1665	ELMSAN1	2
150	COX6C	1	906	LYPLA2	1	1666	ZNF570	2
151	AC243965.1	1	907	POLR2L	1	1667	FOSL1	2
152	TCIRG1	1	908	TMEM156	1	1668	EIF4G2	2
153	FKBP8	1	909	EIF3K	1	1669	DCUN1D3	2
154	PHYKPL	1	910	HAGHL	1	1670	MEF2D	2
155	1-Sep	1	911	NCOR1	1	1671	RSL24D1	2
156	NDUFB2	1	912	BRI3	1	1672	TRAF4	2
157	LCP1	1	913	JMJD8	1	1673	MRPL1	2
158	CD81	1	914	TPR	1	1674	CBX4	2
159	APBB1IP	1	915	VEGFB	1	1675	FABP5	2
160	COTL1	1	916	OSTF1	1	1676	RPL22L1	2
161	AC243960.1	1	917	WDR77	1	1677	NSUN6	2
162	HLA-DQB1	1	918	SH3BGRL	1	1678	STIP1	2
163	S100A11	1	919	UTP25	1	1679	SLC35D1	2
164	SYNGR1	1	920	ORMDL2	1	1680	GLS	2
165	SSBP4	1	921	PKN1	1	1681	SNU13	2
166	UQCR10	1	922	PEPD	1	1682	NDEL1	2
167	ITM2A	1	923	BABAM1	1	1683	WDR47	2
168	COG2	1	924	DNAJC1	1	1684	SPATA2L	2
169	MRPS34	1	925	G6PD	1	1685	AC058791.1	2
170	MATK	1	926	SDHAF2	1	1686	TARS	2
171	IFITM3	1	927	NUDCD2	1	1687	NUDT4	2
172	PPP1R18	1	928	PARP16	1	1688	SERPINH1	2
173	UBE2L6	1	929	SRP72	1	1689	LMNB1	2
174	APRT	1	930	LZTR1	1	1690	SS18L2	2
175	GSTK1	1	931	TMA16	1	1691	IL23R	2
176	TESC	1	932	GNG5	1	1692	UBR1	2
177	YWHAB	1	933	NAPEPLD	1	1693	SLC25A3	2
178	SLFN5	1	934	C19orf53	1	1694	CD27	2
179	RASA1	1	935	MTERF4	1	1695	TMEM88	2
180	NEDD8	1	936	CUTA	1	1696	DNTTIP2	2
181	DYNLT1	1	937	VSTM4	1	1697	STMN1	2
182	AP3S1	1	938	NAXE	1	1698	LETM2	2
183	PARP8	1	939	AC110769.2	1	1699	MASTL	2
184	AC023157.3	1	940	MIF4GD	1	1700	UBE2D1	2
185	ATP5F1A	1	941	MZT2A	1	1701	CHIC2	2
186	ARPC5	1	942	PDLIM1	1	1702	AL021707.6	2
187	ATP5F1D	1	943	NELFB	1	1703	MAZ	2
188	RNF187	1	944	CUEDC2	1	1704	TOP1	2
189	LAMTOR4	1	945	EXOSC7	1	1705	SPRY1	2
190	COPE	1	946	FGD4	1	1706	VEGFA	2
191	PLEKHJ1	1	947	SNRPD3	1	1707	TP53INP2	2
192	TMED9	1	948	POLR2J	1	1708	CYLD	2
193	EID1	1	949	STK17A	1	1709	FAM222A	2
194	NDUFC2	1	950	MESD	1	1710	PDCL3	2
195	ANP32A	1	951	RASAL3	1	1711	INPP4B	2
196	PSMB1	1	952	MEA1	1	1712	TESK1	2
197	PSMB9	1	953	DYNC1I2	1	1713	ALB	2
198	ATP5MC3	1	954	VPS36	1	1714	STARD7	2
199	ATP5ME	1	955	ABHD14B	1	1715	RNF138	2
200	MMP25-AS	1	956	MRPL44	1	1716	PER2	2
201	FBXW5	1	957	RNF167	1	1717	AHI1	2
202	IPCEF1	1	958	CCDC127	1	1718	AC009404.1	2

203	TGFBR2	1
204	PEBP1	1
205	PKM	1
206	TPI1	1
207	IGHG1	1
208	SNTB2	1
209	LAMTOR1	1
210	LINC00324	1
211	MIEN1	1
212	LAPTM5	1
213	CALM3	1
214	CCT7	1
215	NME1	1
216	RFLNB	1
217	COX8A	1
218	C19orf70	1
219	RSBN1L	1
220	DERL1	1
221	POLR2G	1
222	ATP5MPL	1
223	BCAP31	1
224	TGOLN2	1
225	UBL5	1
226	HBB	1
227	ATP5MF	1
228	RNF213	1
229	CEBPD	1
230	HMOX2	1
231	LINC01970	1
232	CHMP2A	1
233	COPS9	1
234	NUCKS1	1
235	ACTR2	1
236	FERMT3	1
237	FIS1	1
238	NABP1	1
239	CSTB	1
240	TRBC2	1
241	6-Sep	1
242	HIST1H2BN	1
243	ADH5	1
244	PSMB10	1
245	H1FX	1
246	AP2M1	1
247	MCTP2	1
248	ACTR3	1
249	SDF2L1	1
250	COQ7	1
251	ATP5PO	1
252	ETFB	1
253	TSTD1	1
254	ATP6V0E1	1
255	AL645728.1	1
256	DBNL	1
257	DENND2D	1
258	TMEM109	1
259	NDUFA3	1
260	IGFBP2	1
261	GNAI2	1
262	AL135925.1	1
263	SRM	1
264	TMEM230	1
265	APOBEC3G	1
266	HNRNPD	1
267	MZT2B	1
268	HCLS1	1
269	PPP1R2C	1
270	BSG	1
959	PRR13	1
960	PSIP1	1
961	SH3GLB2	1
962	TMEM258	1
963	MGAT4B	1
964	SUMF2	1
965	SDHAF1	1
966	SRSF10	1
967	HEXDC	1
968	TAF15	1
969	OTUD6B-A	1
970	VKORC1	1
971	C14orf119	1
972	FO393401.1	1
973	LINC02084	1
974	TMEM134	1
975	TBCE	1
976	DCTPP1	1
977	TFB1M	1
978	EPS8L2	1
979	EMC6	1
980	AC026471.1	1
981	PPIE	1
982	CRAMP1	1
983	THUMPD3	1
984	AKT1	1
985	MIGA1	1
986	SEC22B	1
987	DENND6A-	1
988	ADGRG3	1
989	LPIN2	1
990	NAP1L4	1
991	ZNF326	1
992	AL358472.4	1
993	FANCF	1
994	MAPKAPK	1
995	STXBP2	1
996	HIGD1A	1
997	GYPC	1
998	COA1	1
999	TMEM179B	1
1000	VPS13C	1
1001	FEZ2	1
1002	RNPC3	1
1003	SRSF8	1
1004	JCHAIN	1
1005	KLRF1	1
1006	TMEM256	1
1007	RBM25	1
1008	FYN	1
1009	ARL16	1
1010	DRG2	1
1011	LUC7L3	1
1012	ANGEL2	1
1013	AP1M1	1
1014	SPSB3	1
1015	CCDC115	1
1016	PLD3	1
1017	AC099778.1	1
1018	AC012360.3	1
1019	SEM1	1
1020	VTI1B	1
1021	THOC7	1
1022	AK9	1
1023	PPCS	1
1024	MRPS33	1
1025	PCYT2	1
1026	ATP5PB	1
1719	SNHG15	2
1720	UBAP1	2
1721	INTS6L	2
1722	CYGB	2
1723	CH25H	2
1724	B3GALNT2	2
1725	ADRA2B	2
1726	PHLDB2	2
1727	SEC24A	2
1728	CDC37	2
1729	CTDP1	2
1730	CCNYL1	2
1731	ATF4	2
1732	ARL4D	2
1733	DUSP10	2
1734	KCNQ1OT1	2
1735	KLF16	2
1736	ARHGEF39	2
1737	SPAG4	2
1738	EZH2	2
1739	STAG2	2
1740	F12	2
1741	MED13	2
1742	RAB11FIP1	2
1743	EML4	2
1744	RNMT	2
1745	IRGQ	2
1746	GEM	2
1747	TENT4B	2
1748	WDR26	2
1749	TGIF1	2
1750	WDR45B	2
1751	NFAT5	2
1752	IL27RA	2
1753	HELB	2
1754	ARID4B	2
1755	SKI	2
1756	CGAS	2
1757	RNF125	2
1758	BRMS1L	2
1759	EIF3J	2
1760	CCL20	2
1761	HBP1	2
1762	BRD1	2
1763	RPL8	2
1764	PNP	2
1765	NRBF2	2
1766	SESN2	2
1767	CXCL2	2
1768	CYB5D1	2
1769	WHRN	2
1770	TRA2B	2
1771	STAT4	2
1772	S1PR2	2
1773	RBBP6	2
1774	MAGOH	2
1775	RHOB	2
1776	STX17-AS1	2
1777	N4BP2L1	2
1778	PLEKHA2	2
1779	LTA	2
1780	LYSMD3	2
1781	IL1R2	2
1782	MRFAP1	2
1783	HIST1H1A	2
1784	LINC00513	2
1785	KANSL2	2
1786	AC144831.1	2

271	TWF2	1	1027	TMX3	1	1787	DRAM1	2
272	RBM3	1	1028	MBD2	1	1788	RBM12	2
273	TMED4	1	1029	PPP1R3E	1	1789	SNAPC1	2
274	ABI3	1	1030	PDZD11	1	1790	BEX5	2
275	BRK1	1	1031	SLC35B1	1	1791	CXorf40A	2
276	SMC1A	1	1032	PMS1	1	1792	TXLNG	2
277	PSMA5	1	1033	RNASEH2C	1	1793	G0S2	2
278	DDX46	1	1034	EIF4A2	1	1794	CD44	2
279	CD74	1	1035	TMEM14C	1	1795	NARF	2
280	PYCARD	1	1036	SPATC1L	1	1796	C2orf40	2
281	PHPT1	1	1037	PEX13	1	1797	PPP2R2A	2
282	MINOS1	1	1038	HIPK3	1	1798	NSD3	2
283	CFLAR	1	1039	AAK1	1	1799	ALAS1	2
284	PIP4K2A	1	1040	BOD1L1	1	1800	SPAG1	2
285	PREX1	1	1041	CBWD3	1	1801	DDX18	2
286	PRTN3	1	1042	THEMIS2	1	1802	INSIG1	2
287	SP100	1	1043	AL391121.1	1	1803	MYLIP	2
288	ATP5PF	1	1044	CABIN1	1	1804	HES1	2
289	ATP5MC1	1	1045	SAP18	1	1805	PAF1	2
290	IL18	1	1046	TLK1	1	1806	CAAP1	2
291	AC012306.2	1	1047	SMG6	1	1807	RGS13	2
292	IKZF3	1	1048	STOM	1	1808	AC016831.1	2
293	C9orf16	1	1049	PPP1CC	1	1809	COX7B	2
294	MAP4	1	1050	MRPS12	1	1810	CRY1	2
295	SNX17	1	1051	VAMP4	1	1811	BUD31	2
296	IL2RB	1	1052	H2AFY	1	1812	PTS	2
297	RPA2	1	1053	LYRM2	1	1813	RNF19B	2
298	VAMP2	1	1054	HACL1	1	1814	AL021707.1	2
299	AL021453.1	1	1055	DDX39B	1	1815	ASTE1	2
300	PSMB8-AS1	1	1056	ATP6AP2	1	1816	RLF	2
301	ABRACL	1	1057	LINC00847	1	1817	SYTL3	2
302	EPS8	1	1058	PIM1	1	1818	AL731571.1	2
303	MPHOSPH8	1	1059	SLAMF6	1	1819	BAIAP2	2
304	NCR1	1	1060	PPM1G	1	1820	CKAP2	2
305	RCSD1	1	1061	UBASH3B	1	1821	HNRNPU	2
306	GNPTAB	1	1062	TOMM40	1	1822	TUFT1	2
307	DRAP1	1	1063	LINC00476	1	1823	PPP1R13B	2
308	TMBIM4	1	1064	CRELD2	1	1824	EIF4A1	2
309	STMP1	1	1065	GART	1	1825	ZRANB1	2
310	COX7A2L	1	1066	TMEM161B	1	1826	SREBF1	2
311	H3F3A	1	1067	SRSF4	1	1827	SLC45A4	2
312	TRGC1	1	1068	ERV3-1	1	1828	HP	2
313	KLRC4	1	1069	MX1	1	1829	RBMX	2
314	LAMTOR2	1	1070	Z93930.2	1	1830	HIST1H3B	2
315	RPP38	1	1071	PSMD3	1	1831	MED31	2
316	MRPL51	1	1072	QTRT1	1	1832	STRAP	2
317	RAD23A	1	1073	ZNF429	1	1833	UBE2H	2
318	WAS	1	1074	C11orf71	1	1834	RPP21	2
319	TXN2	1	1075	MFNG	1	1835	WAC	2
320	PNN	1	1076	ARHGEF1	1	1836	APOA2	2
321	PARP1	1	1077	SEPHS2	1	1837	PHF1	2
322	TMEM14B	1	1078	SLC16A1-A	1	1838	SHOC2	2
323	COX5B	1	1079	DEF6	1	1839	ZNF165	2
324	GFI1	1	1080	TTC13	1	1840	CXCL8	2
325	ZNF844	1	1081	NDUFB5	1	1841	YTHDC1	2
326	SKAP1	1	1082	PILRB	1	1842	HSPA5	2
327	HMGN2	1	1083	STX7	1	1843	APOA1	2
328	NDUFB3	1	1084	LBH	1	1844	SMARCA5	2
329	PPAN	1	1085	AAMP	1	1845	CDK5RAP1	2
330	ARPC1B	1	1086	CASP2	1	1846	CHKA	2
331	ITGB1BP1	1	1087	ZNF83	1	1847	ZPR1	2
332	TUFM	1	1088	RPS19BP1	1	1848	REV3L	2
333	PHB2	1	1089	CIDEB	1	1849	MIR194-2H	2
334	DYNLRB1	1	1090	SCML4	1	1850	AC104116.1	2
335	ZNHIT6	1	1091	RMRP	1	1851	EIF1AX	2
336	GOLGA8A	1	1092	COMMD10	1	1852	GPM6B	2
337	NDUFAF3	1	1093	POLDIP2	1	1853	HIVEP2	2
338	TBCB	1	1094	C6orf226	1	1854	CCDC59	2

339	AC020911.2	1	1095	UNG	1	1855	ZNF706	2
340	CELF2	1	1096	UQCRC2	1	1856	ABCF1	2
341	FADD	1	1097	COX18	1	1857	DSCR9	2
342	PYCARD-A	1	1098	GPANK1	1	1858	SNRPB	2
343	AC005332.5	1	1099	FOPNL	1	1859	ZNF10	2
344	FAM45A	1	1100	DNAJC4	1	1860	RAB1A	2
345	TIMM8B	1	1101	GPSM3	1	1861	H2AFX	2
346	TRIM14	1	1102	ANAPC4	1	1862	AC026979.2	2
347	ADM	1	1103	SNRNP200	1	1863	ERO1B	2
348	THRAP3	1	1104	AC027644.3	1	1864	SOCS4	2
349	P4HB	1	1105	LINC00891	1	1865	KMT5C	2
350	SRP14	1	1106	SPA17	1	1866	ELOC	2
351	SNRNP70	1	1107	AL136040.1	1	1867	RAB5A	2
352	EDF1	1	1108	HTATIP2	1	1868	UQCRH	2
353	CAPN1	1	1109	ARL17A	1	1869	EFNB2	2
354	HPGD	1	1110	MCTS1	1	1870	AC124798.1	2
355	COPZ1	1	1111	HDDC3	1	1871	JMY	2
356	SMDT1	1	1112	BRD9	1	1872	FXR1	2
357	ZAP70	1	1113	UBE2G2	1	1873	TCF7	2
358	ERAP2	1	1114	MYBL1	1	1874	GPR35	2
359	GIMAP6	1	1115	RHOBTB3	1	1875	WSB1	2
360	LSM7	1	1116	CTSA	1	1876	AHR	2
361	METTL9	1	1117	NDUFA13	1	1877	FEZ1	2
362	NME3	1	1118	HDAC10	1	1878	CERK	2
363	ARPC5L	1	1119	LMF2	1	1879	ATF3	3
364	ZMAT2	1	1120	FAAP20	1	1880	CCL4	3
365	TNFRSF1A	1	1121	SIRT7	1	1881	CCL4L2	3
366	NEMF	1	1122	CDK5RAP3	1	1882	TNFAIP3	3
367	ATP5F1B	1	1123	CASP6	1	1883	GRASP	3
368	POLR2E	1	1124	MRPL52	1	1884	KLF2	3
369	SSR4	1	1125	GPR174	1	1885	CREM	3
370	TMBIM6	1	1126	ZCRB1	1	1886	NFE2L2	3
371	FNBP4	1	1127	USP48	1	1887	GNLY	3
372	GUK1	1	1128	TRIM13	1	1888	KLF6	3
373	CYB5D2	1	1129	AZI2	1	1889	PTGER4	3
374	COA3	1	1130	NDUFS3	1	1890	TXNIP	3
375	MYDGF	1	1131	NIT2	1	1891	NKG7	3
376	TIA1	1	1132	SRP9	1	1892	BHLHE40	3
377	COMMD6	1	1133	CTSG	1	1893	PMAIP1	3
378	PA2G4	1	1134	YARS	1	1894	HLA-E	3
379	ZNF160	1	1135	AC093462.1	1	1895	CRIP1	3
380	MDFIC	1	1136	PDCD2	1	1896	ISG20	3
381	SNX10	1	1137	RIPK3	1	1897	RPS4Y1	3
382	SSH2	1	1138	AKAP12	1	1898	CD3E	3
383	PPP1R7	1	1139	OGA	1	1899	ZFP36L2	3
384	TALDO1	1	1140	SNRPC	1	1900	ANXA1	3
385	CTDSP1	1	1141	PDLIM2	1	1901	PER1	3
386	7-Sep	1	1142	PTPN18	1	1902	SQSTM1	3
387	ZBTB38	1	1143	TRNAU1AP	1	1903	RPS3A	3
388	TRNT1	1	1144	AC245014.3	1	1904	PRF1	3
389	ATP5MG	1	1145	ORMDL1	1	1905	FTH1	3
390	IQGAP1	1	1146	TRIP11	1	1906	RGS2	3
391	KLHL6	1	1147	EIF3I	1	1907	CYBA	3
392	PHB	1	1148	SLA2	1	1908	IL32	3
393	BLOC1S1	1	1149	AC142472.1	1	1909	RPL14	3
394	DMAC1	1	1150	ADAM28	1	1910	MT-CYB	3
395	ORAI2	1	1151	ARFGAP2	1	1911	HLA-C	3
396	ATF6B	1	1152	C9orf64	1	1912	RILPL2	3
397	ICAM3	1	1153	DDX43	1	1913	CYTIP	3
398	CXorf38	1	1154	NUDT16L1	1	1914	VIM	3
399	EIF3J-DT	1	1155	OTUB1	1	1915	SH3BGRL3	3
400	NAA10	1	1156	PGP	1	1916	TAGLN2	3
401	SF3B2	1	1157	PIGS	1	1917	BRD2	3
402	IFI27L2	1	1158	PPOX	1	1918	GPR65	3
403	TADA3	1	1159	ST3GAL1	1	1919	IER3	3
404	NUCB1	1	1160	C8orf33	1	1920	CTSW	3
405	ATRAID	1	1161	ENOPH1	1	1921	PIM3	3
406	BANF1	1	1162	GTF2A2	1	1922	GZMH	3

407	GPAA1	1	1163	SNX14	1	1923	HSPB1	3
408	UQCRQ	1	1164	ARFIP2	1	1924	RPS4X	3
409	DGCR6L	1	1165	CTBS	1	1925	RUNX3	3
410	NDUFB8	1	1166	RAD9A	1	1926	SAT1	3
411	CMTM3	1	1167	POP4	1	1927	LMNA	3
412	FAM50A	1	1168	MUS81	1	1928	CD2	3
413	RANGRF	1	1169	CHMP1A	1	1929	CDKN1A	3
414	HP1BP3	1	1170	URI1	1	1930	IVNS1ABP	3
415	CISD3	1	1171	ZNF253	1	1931	NEAT1	3
416	TMEM223	1	1172	RTL8A	1	1932	CD55	3
417	HNRNPA3	1	1173	LSM8	1	1933	PHLDA1	3
418	ILK	1	1174	DPYSL2	1	1934	PRDM1	3
419	ANKRD36	1	1175	CSRP1	1	1935	TMSB10	3
420	PCSK7	1	1176	ZDHHC12	1	1936	MTRNR2L1	3
421	POLR3GL	1	1177	AP001462.1	1	1937	UAP1	3
422	ITM2B	1	1178	ATXN7L3B	1	1938	ITGB7	3
423	NDUFA12	1	1179	PPP1R3D	1	1939	RPL7A	3
424	CHURC1	1	1180	CLASP1	1	1940	MT-CO2	3
425	CYBC1	1	1181	ATL3	1	1941	UCP2	3
426	PTPN12	1	1182	KDELR1	1	1942	RPS21	3
427	CCNL2	1	1183	NSUN5	1	1943	CD3D	3
428	XRCC6	1	1184	R3HCC1	1	1944	PPP1R10	3
429	YPEL1	1	1185	NUDCD3	1	1945	LITAF	3
430	RAB30-AS1	1	1186	LIG1	1	1946	RPL28	3
431	SCP2	1	1187	CLINT1	1	1947	CARD16	3
432	NDUFS2	1	1188	SDHD	1	1948	TMEM173	3
433	RAP1GDS1	1	1189	PCNX1	1	1949	SLFN11	3
434	AC083973.1	1	1190	HGH1	1	1950	RPS5	3
435	VPS28	1	1191	ERGIC2	1	1951	NEU1	3
436	B4GALT4	1	1192	SF3A3	1	1952	RNF19A	3
437	MRPL57	1	1193	UGP2	1	1953	RPL21	3
438	ZRANB2	1	1194	KIN	1	1954	TNF	3
439	UROS	1	1195	YIF1A	1	1955	SH3BP5	3
440	ARHGEF9	1	1196	RNPEP	1	1956	HOPX	3
441	GATAD1	1	1197	UBE2L3	1	1957	KLF13	3
442	PSMA1	1	1198	RPUSD3	1	1958	GNG2	3
443	TXN	1	1199	TMEM183A	1	1959	S100A4	3
444	ATP5PD	1	1200	ASCC1	1	1960	CST7	3
445	ELANE	1	1201	OARD1	1	1961	CD3G	3
446	ZNHIT1	1	1202	COA6	1	1962	RPS27	3
447	FKBP1A	1	1203	TBC1D1	1	1963	B2M	3
448	TTC14	1	1204	VMP1	1	1964	SPON2	3
449	PSMD8	1	1205	DNPEP	1	1965	AES	3
450	LSM10	1	1206	PRPSAP2	1	1966	TLE4	3
451	NDUFV1	1	1207	YIPF2	1	1967	RPS14	3
452	RBCK1	1	1208	ZNF701	1	1968	RPS9	3
453	GCHFR	1	1209	ARHGAP30	1	1969	TNFRSF18	3
454	BCL7C	1	1210	ZNF708	1	1970	RPL39	3
455	B3GALT4	1	1211	DBI	1	1971	ANXA2	3
456	COX4I1	1	1212	NHLRC2	1	1972	COQ10B	3
457	TMEM101	1	1213	LEPROT	1	1973	MT-CO1	3
458	CREBZF	1	1214	LINC00685	1	1974	ADGRG1	3
459	CCM2	1	1215	LRPAP1	1	1975	RPSA	3
460	SNF8	1	1216	C18orf21	1	1976	MLF1	3
461	NDUFS6	1	1217	EIF2B1	1	1977	COX7C	3
462	GYG1	1	1218	SMARCE1	1	1978	SNRPD2	3
463	GPS1	1	1219	AL137077.2	1	1979	TNFRSF1B	3
464	NHP2	1	1220	PRPF38B	1	1980	BAZ1A	3
465	HNRNPA2E	1	1221	CEBPZOS	1	1981	NDUFB7	3
466	MRPL41	1	1222	MPPE1	1	1982	S100A6	3
467	TAPBP	1	1223	MRPL49	1	1983	RPS3	3
468	CPNE1	1	1224	IWS1	1	1984	AC091271.1	3
469	SLC25A11	1	1225	YPEL3	1	1985	EMP3	3
470	AP2S1	1	1226	ARIH2OS	1	1986	BX284668.6	3
471	MAGED2	1	1227	RBM6	1	1987	FCGR3A	3
472	TMEM59	1	1228	PSMD13	1	1988	MRPS6	3
473	DPH7	1	1229	RTCB	1	1989	SLC5A3	3
474	ANAPC16	1	1230	CRELD1	1	1990	MBP	3

475	ISCU	1	1231	CPT2	1	1991	PTPN6	3
476	P2RX5	1	1232	PSME3	1	1992	AC087239.1	3
477	EAPP	1	1233	SNX3	1	1993	MIR22HG	3
478	HIST1H2BH	1	1234	ZNF688	1	1994	RPS15	3
479	NDUFA11	1	1235	TMEM9B	1	1995	RPL13	3
480	PGLS	1	1236	OCIAD2	1	1996	BAG3	3
481	NCBP2-AS2	1	1237	EIF2B2	1	1997	ID3	3
482	ARHGAP4	1	1238	KLHL23	1	1998	RPS25	3
483	MRPS18B	1	1239	RNF181	1	1999	STK4	3
484	COPS6	1	1240	NDUFB11	1	2000	RPS29	3
485	MZB1	1	1241	IFRD2	1	2001	RPL18A	3
486	NCK2	1	1242	PAFAH2	1	2002	RPL19	3
487	RPN2	1	1243	GTF3C5	1	2003	LDHB	3
488	UBL4A	1	1244	NFATC2IP	1	2004	SMAP2	3
489	MAPK13	1	1245	ASB8	1	2005	BIN1	3
490	S100PBP	1	1246	GEMIN6	1	2006	LIMD2	3
491	TSPAN31	1	1247	RGPD2	1	2007	IL7R	3
492	TMEM219	1	1248	SCAPER	1	2008	OSM	3
493	SERPINB6	1	1249	MTERF3	1	2009	AVPI1	3
494	SDHC	1	1250	TTLL3	1	2010	RPL37	3
495	FYB1	1	1251	C16orf58	1	2011	RPL29	3
496	NDUFS8	1	1252	WDR61	1	2012	CD8A	3
497	CCNDBP1	1	1253	COMMD8	1	2013	CD8B	3
498	FBXO2	1	1254	AL606760.3	1	2014	RPS15A	3
499	PPIH	1	1255	URM1	1	2015	RPL6	3
500	MCRIP1	1	1256	AC064807.1	1	2016	TNFRSF9	3
501	KLRB1	1	1257	DDX28	1	2017	CDC42SE1	3
502	MRPS23	1	1258	METTL18	1	2018	DUSP8	3
503	PDIA3	1	1259	RNF130	1	2019	GZMB	3
504	RPE	1	1260	PFDN2	1	2020	LDLR	3
505	C11orf68	1	1261	RAC1	1	2021	RPL23A	3
506	TRAPPC6A	1	1262	NECAP2	1	2022	ARL4C	3
507	COX7A2	1	1263	MBIP	1	2023	ERRFI1	3
508	CTSZ	1	1264	SRSF1	1	2024	ITPRIP	3
509	ZNF224	1	1265	PPM1M	1	2025	CCND3	3
510	AKR1B1	1	1266	SCCPDH	1	2026	MT-ND1	3
511	SRI	1	1267	FUCA1	1	2027	RACK1	3
512	AGTRAP	1	1268	VPS4B	1	2028	HLA-DRB1	3
513	HNRNPR	1	1269	NKILA	1	2029	TTC38	3
514	ARFRP1	1	1270	RAB37	1	2030	SOX4	3
515	MRPL23	1	1271	CENPX	1	2031	RPL10	3
516	MED7	1	1272	ESF1	1	2032	RABAC1	3
517	LAMP1	1	1273	MRPS14	1	2033	RPS28	3
518	NDUFA9	1	1274	COA5	1	2034	TPST2	3
519	NCAM1	1	1275	ANXA11	1	2035	HSH2D	3
520	C9orf78	1	1276	ACP1	1	2036	RPL35A	3
521	GFOD1	1	1277	CTSC	1	2037	RPL27A	3
522	SRSF11	1	1278	RWDD1	1	2038	HEXIM1	3
523	CHMP4A	1	1279	MZF1-AS1	1	2039	TMEM71	3
524	LENG8	1	1280	AL031708.1	1	2040	CASP8	3
525	EPSTI1	1	1281	MED8	1	2041	OAT	3
526	SMIM7	1	1282	SNRPA1	1	2042	RPL30	3
527	CNBP	1	1283	MIR4435-2H	1	2043	KLRG1	3
528	SELENOH	1	1284	TNFSF10	1	2044	PPDPF	3
529	PRSS21	1	1285	ELP5	1	2045	JAK1	3
530	METTL17	1	1286	PPP1R12A	1	2046	ATP2B1-AS	3
531	LINC02256	1	1287	AC137767.1	1	2047	RPS24	3
532	ACAA2	1	1288	ABHD11	1	2048	RPL34	3
533	GRK2	1	1289	C22orf39	1	2049	ZC3HAV1	3
534	IGHG4	1	1290	PIN1	1	2050	DDIT3	3
535	PLA2G16	1	1291	AP4B1	1	2051	MT-ATP6	3
536	DPM2	1	1292	SELENOT	1	2052	SELPLG	3
537	PSMC5	1	1293	ELF2	1	2053	ARPC2	3
538	TNFSF12	1	1294	SPNS3	1	2054	CD52	3
539	REEP5	1	1295	LINC02001	1	2055	SAMHD1	3
540	DCAF7	1	1296	C2orf68	1	2056	RASGRP2	3
541	OSTC	1	1297	KEAP1	1	2057	MALAT1	3
542	RCCD1	1	1298	CHKB	1	2058	CAPG	3

543	PYURF	1	1299	DNASE1	1	2059	PRDX2	3
544	FAM173A	1	1300	SSU72	1	2060	NCR3	3
545	IAH1	1	1301	B3GAT3	1	2061	TTTY15	3
546	AC025164.1	1	1302	NME6	1	2062	RPLP2	3
547	GON4L	1	1303	AREG	2	2063	LGALS1	3
548	AL355472.1	1	1304	ATP1B3	2	2064	HIF1A	3
549	APOBEC3C	1	1305	CD69	2	2065	PPIA	3
550	RPL7L1	1	1306	DNAJA1	2	2066	XBP1	3
551	SHISA5	1	1307	DNAJB1	2	2067	SGK1	3
552	CD151	1	1308	DNAJB6	2	2068	RPS16	3
553	ACP5	1	1309	DUSP1	2	2069	MT-ND4	3
554	TLE1	1	1310	DUSP2	2	2070	RPS19	3
555	AC245297.3	1	1311	FAM177A1	2	2071	UPP1	3
556	SUPT16H	1	1312	FOSB	2	2072	RPL10A	3
557	HENMT1	1	1313	HSP90AA1	2	2073	RPS18	3
558	CDK2AP2	1	1314	HSP90AB1	2	2074	SYNE2	3
559	IL16	1	1315	HSPA1A	2	2075	LINC00861	3
560	TSR2	1	1316	HSPA1B	2	2076	ULBP2	3
561	PRDX6	1	1317	HSPA8	2	2077	SOD1	3
562	TRIM73	1	1318	HSPD1	2	2078	GLA	3
563	COX17	1	1319	HSPE1	2	2079	GBP5	3
564	RCN2	1	1320	HSPH1	2	2080	TRBC1	3
565	EIF2S3	1	1321	IRF1	2	2081	Z93241.1	3
566	CRACR2B	1	1322	JUN	2	2082	IQCN	3
567	PSMD4	1	1323	JUNB	2	2083	NACA	3
568	FGR	1	1324	JUND	2	2084	CLEC2B	3
569	TMEM140	1	1325	METRNL	2	2085	C12orf57	3
570	P2RY11	1	1326	NFKBIA	2	2086	MT-CO3	3
571	NUTM2B-A	1	1327	NR4A1	2	2087	SUN2	3
572	PGAM1	1	1328	NR4A2	2	2088	MT2A	3
573	SURF6	1	1329	PABPC1	2	2089	SSR2	3
574	NUBP2	1	1330	PDE4B	2	2090	UTY	3
575	DGUOK	1	1331	PPP1R15A	2	2091	CLCF1	3
576	TAF8	1	1332	REL	2	2092	BIN2	3
577	IFI44	1	1333	RGCC	2	2093	GLIPR2	3
578	COA4	1	1334	TIPARP	2	2094	RPS8	3
579	HMGB2	1	1335	TWISTNB	2	2095	LCK	3
580	RFC1	1	1336	UBB	2	2096	S1PR5	3
581	KRT10	1	1337	YPEL5	2	2097	RPL11	3
582	KRT81	1	1338	ZFP36	2	2098	RPS23	3
583	SUGP2	1	1339	ZNF331	2	2099	RPL37A	3
584	IARS2	1	1340	CD83	2	2100	UBA52	3
585	LAT	1	1341	CHMP1B	2	2101	MTHFD2	3
586	TRABD	1	1342	HSPA6	2	2102	MT1X	3
587	AC005837.1	1	1343	UBC	2	2103	AL450998.2	3
588	SSNA1	1	1344	CEMIP2	2	2104	KLF9	3
589	PSMA7	1	1345	NFKBIZ	2	2105	RPL36AL	3
590	C19orf25	1	1346	ARL5B	2	2106	KMT2E-AS1	3
591	THUMPD2	1	1347	ARL4A	2	2107	CDC42EP3	3
592	FIBP	1	1348	KDM6B	2	2108	MAPRE2	3
593	GSDMD	1	1349	CCL3	2	2109	TRGV4	3
594	SNRPA	1	1350	BTG2	2	2110	RPL26	3
595	TPM3	1	1351	MCL1	2	2111	BPGM	3
596	COX6A1	1	1352	NR4A3	2	2112	CD300A	3
597	SPAG7	1	1353	CSRNP1	2	2113	AC074044.1	3
598	CYC1	1	1354	ICAM1	2	2114	RPL41	3
599	PWWP2A	1	1355	SELENOK	2	2115	SYTL1	3
600	HEBP2	1	1356	TUBA4A	2	2116	ADRB2	3
601	MDM4	1	1357	TNFSF9	2	2117	MPV17	3
602	NKTR	1	1358	CXCR4	2	2118	RPS27A	3
603	PNKP	1	1359	UBE2S	2	2119	HNRNPL	3
604	GUSB	1	1360	CHORDC1	2	2120	AL139274.2	3
605	TSPO	1	1361	TSC22D3	2	2121	INTS6-AS1	3
606	CCS	1	1362	IFNG	2	2122	PRMT5-AS1	3
607	MRPL43	1	1363	MAFF	2	2123	RPL38	3
608	NDUFAF8	1	1364	JMJD6	2	2124	HLA-DRA	3
609	DUSP23	1	1365	H2AFZ	2	2125	KLRC2	3
610	ALDH9A1	1	1366	IFRD1	2	2126	MT-ND2	3

611	PDIA6	1	1367	DDX3Y	2	2127	MFSD10	3
612	ZNF91	1	1368	FOS	2	2128	MT-ND5	3
613	CSNK2B	1	1369	NFKB1	2	2129	FTL	3
614	ZNF652	1	1370	ZFAND5	2	2130	RPL9	3
615	BCO2	1	1371	SPTY2D1	2	2131	SNHG25	3
616	CAPNS1	1	1372	RGS1	2	2132	TOMM7	3
617	SLC25A45	1	1373	IDI1	2	2133	YWHAQ	3
618	DHRS3	1	1374	SERTAD1	2	2134	HLA-DPB1	3
619	ANKRD36C	1	1375	FOSL2	2	2135	ADAP1	3
620	CORO1B	1	1376	SKIL	2	2136	TRAC	3
621	PSMD7	1	1377	CCNH	2	2137	MT-ATP8	3
622	TP53TG1	1	1378	RASGEF1B	2	2138	SLA	3
623	TSTA3	1	1379	ELF1	2	2139	LACTB	3
624	UQCC3	1	1380	ZC3H12A	2	2140	PTPN4	3
625	TMEM273	1	1381	CACYBP	2	2141	SP140	3
626	TMED2	1	1382	VPS37B	2	2142	B4GALT1	3
627	HDAC3	1	1383	CKS2	2	2143	ABHD17A	3
628	USP28	1	1384	PPP1R2	2	2144	PFDN5	3
629	IDH2	1	1385	MYADM	2	2145	RPL36A	3
630	SELENOF	1	1386	MRPL18	2	2146	LYST	3
631	PYCR2	1	1387	DUSP5	2	2147	STAT3	3
632	DPP7	1	1388	RANBP2	2	2148	AHNAK	3
633	CDK5R1	1	1389	PIK3R1	2	2149	RGS19	3
634	2-Mar	1	1390	ARF4	2	2150	AC023509.4	3
635	REX1BD	1	1391	ELL2	2	2151	NFKBIB	3
636	DDX17	1	1392	EGR1	2	2152	MLLT11	3
637	SH2D1A	1	1393	DYNLL1	2	2153	U2AF1	3
638	TRIM44	1	1394	EIF1	2	2154	AKAP13	3
639	SNRPE	1	1395	H3F3B	2	2155	GLRX	3
640	MDH1	1	1396	XCL2	2	2156	MTRNR2L1	3
641	PDHB	1	1397	IER5	2	2157	DOK1	3
642	GIMAP2	1	1398	MAP3K8	2	2158	ZFP36L1	3
643	GLOD4	1	1399	SCX	2	2159	RPS7	3
644	LMAN2	1	1400	SYAP1	2	2160	AC009812.1	3
645	CCR1	1	1401	ID1	2	2161	RPL36	3
646	CNPY3	1	1402	TPT1	2	2162	AZU1	3
647	OXNAD1	1	1403	ANKRD37	2	2163	CD37	3
648	PFKL	1	1404	LDHA	2	2164	EIF3D	3
649	NDUFA8	1	1405	NAF1	2	2165	NDUFS7	3
650	TSEN54	1	1406	EIF4A3	2	2166	CIB1	3
651	WDR74	1	1407	ZBTB1	2	2167	GPR108	3
652	HEXA	1	1408	GTF2B	2	2168	JAKMIP1	3
653	POLM	1	1409	SRSF2	2	2169	FGFBP2	3
654	WASHC1	1	1410	ETV3	2	2170	TBRG1	3
655	AKR7A2	1	1411	NINJ1	2	2171	MRPS21	3
656	AIP	1	1412	SARAF	2	2172	RPL27	3
657	ACADVL	1	1413	HES4	2	2173	CAPN2	3
658	JPX	1	1414	IRF8	2	2174	ARHGEF37	3
659	TMED10	1	1415	PLIN2	2	2175	RPS12	3
660	TCEAL8	1	1416	RAB8B	2	2176	ZMYND8	3
661	CDK9	1	1417	AC020916.1	2	2177	ANP32B	3
662	CDK10	1	1418	GNL3	2	2178	ZBP1	3
663	PRPF31	1	1419	NXT1	2	2179	PLK2	3
664	LSM3	1	1420	DNAJB4	2	2180	SSBP1	3
665	DUT	1	1421	RALGAPA1	2	2181	SNAI1	3
666	FAM118A	1	1422	PPP1R15B	2	2182	TXNL4A	3
667	CBLB	1	1423	SIK1	2	2183	HAVCR2	3
668	NDUFA1	1	1424	FKBP4	2	2184	HSPA2	3
669	PDIA4	1	1425	PRR7	2	2185	CCDC173	3
670	SCAMP1-AS	1	1426	ZFAND2A	2	2186	RALGDS	3
671	MRPL15	1	1427	STX11	2	2187	RPS11	3
672	PIN4	1	1428	PFKFB3	2	2188	SCLT1	3
673	JTB	1	1429	CCDC107	2	2189	DAZAP2	3
674	SMIM27	1	1430	YWHAZ	2	2190	SAMD3	3
675	SMIM20	1	1431	PTMA	2	2191	ABHD5	3
676	UBE2I	1	1432	CYCS	2	2192	S1PR4	3
677	RNF135	1	1433	BCL2A1	2	2193	BCL11B	3
678	PEF1	1	1434	IER2	2	2194	MX2	3

679	CHMP6	1	1435	GPBP1	2	2195	RPS2	3
680	WASHC3	1	1436	EIF5	2	2196	BTN3A2	3
681	ANAPC11	1	1437	ARIH1	2	2197	DHRS7	3
682	OGG1	1	1438	ADGRE5	2	2198	RND1	3
683	LSM4	1	1439	BCAS2	2	2199	PYM1	3
684	MYH9	1	1440	EZR	2	2200	ANKRD9	3
685	TCTA	1	1441	CALM1	2	2201	HINT1	3
686	N4BP2L2	1	1442	AC044849.1	2	2202	RYK	3
687	VASP	1	1443	NOCT	2	2203	POLR2A	3
688	PPP1R35	1	1444	SDCBP	2	2204	AL356488.3	3
689	YDJC	1	1445	NFIL3	2	2205	CD99	3
690	GADD45GI	1	1446	SRGN	2	2206	RPS6	3
691	PET100	1	1447	STK17B	2	2207	TSPAN32	3
692	SLC2A4RG	1	1448	DCTN6	2	2208	CRABP2	3
693	FBXO22	1	1449	TCP1	2	2209	SLC38A2	3
694	DCAF11	1	1450	ETF1	2	2210	AL451165.2	3
695	PUF60	1	1451	FAM53C	2	2211	ZNF276	3
696	RINL	1	1452	DDX24	2	2212	HNRNPA1	3
697	CLSTN3	1	1453	SLC7A5	2	2213	PRDX1	3
698	UHMK1	1	1454	DDX5	2	2214	POLG2	3
699	ING4	1	1455	NFKBID	2	2215	UBL3	3
700	SELENOW	1	1456	AC016831.7	2	2216	EHD4	3
701	TYMP	1	1457	DDX3X	2	2217	RPL4	3
702	NOP10	1	1458	SOCS3	2	2218	RIPOR2	3
703	ATIC	1	1459	RBBP8	2	2219	C12orf75	3
704	SMC3	1	1460	TSPYL2	2	2220	MRPL16	3
705	ZNF677	1	1461	CHD1	2	2221	PRRG2	3
706	PSMB2	1	1462	DEDD2	2	2222	SELL	3
707	HLA-F	1	1463	BIRC3	2	2223	TMEM238	3
708	AC246785.3	1	1464	HCG18	2	2224	MXD1	3
709	C22orf46	1	1465	CCND2	2	2225	FXYD5	3
710	PDAP1	1	1466	RELB	2	2226	SURF1	3
711	AUP1	1	1467	PTP4A1	2	2227	FAM129A	3
712	DDT	1	1468	ODC1	2	2228	RPL15	3
713	NUDT18	1	1469	BCL3	2	2229	TAP1	3
714	CCDC90B	1	1470	KMT2E	2	2230	TRAT1	3
715	DCTN3	1	1471	NEK1	2	2231	XPA	3
716	RNF146	1	1472	RRAD	2	2232	C7orf50	3
717	NDUFB9	1	1473	BZW1	2	2233	HECA	3
718	ANKRD49	1	1474	KLF10	2	2234	AC106739.2	3
719	COMMD7	1	1475	MARCKSL1	2	2235	AC104695.3	3
720	NMT2	1	1476	DNAJA4	2	2236	RPL22	3
721	NMUR1	1	1477	ANKRD28	2	2237	RPLP0	3
722	AC008105.3	1	1478	IFFO2	2	2238	BAMBI	3
723	TRIM52	1	1479	ERF	2	2239	LINC02446	3
724	CCNT2	1	1480	XCL1	2	2240	C1QA	3
725	ELOVL6	1	1481	BTG1	2	2241	ZRSR2	3
726	DDOST	1	1482	TAGAP	2	2242	TKT	3
727	ZNF43	1	1483	CALM2	2	2243	RPS13	3
728	VAMP5	1	1484	SBDS	2	2244	ZNF584	3
729	AL135791.1	1	1485	HIST1H4C	2	2245	FCGR3B	3
730	MRPL11	1	1486	TENT5C	2	2246	TUBB2A	3
731	ARHGEF10	1	1487	CSF2	2	2247	HLA-DRB5	3
732	CD96	1	1488	CRTAM	2	2248	HAX1	3
733	NMRAL1	1	1489	SAMSN1	2	2249	KLRC3	3
734	GRAMD1C	1	1490	ZNF184	2	2250	PCID2	3
735	CINP	1	1491	YES1	2	2251	RECQL	3
736	NDUFB4	1	1492	NUFIP2	2	2252	HMBOX1	3
737	MRPL40	1	1493	G3BP2	2	2253	DIP2A	3
738	RNPEPL1	1	1494	BRAF	2	2254	SLC27A3	3
739	CCDC66	1	1495	CCL3L1	2	2255	GHDC	3
740	RGL4	1	1496	NPM1	2	2256	LINC02076	3
741	ITGAE	1	1497	ZFY	2	2257	SNHG12	3
742	NDUFC1	1	1498	MIR155HG	2	2258	SLCO3A1	3
743	XPO1	1	1499	OTULIN	2	2259	CRBN	3
744	DCTN2	1	1500	RBM8A	2	2260	NIPSNAP2	3
745	UBLCP1	1	1501	B3GNT7	2	2261	IQGAP2	3
746	TMX4	1	1502	PRMT9	2	2262	ARRB2	3

747	DCTD	1	1503	CCNL1	2	2263	RPL18	3
748	SPG7	1	1504	TPM4	2	2264	DTHD1	3
749	FDPS	1	1505	DOK2	2	2265	SP140L	3
750	ANAPC15	1	1506	RGS16	2	2266	ERP29	3
751	DENR	1	1507	JOSD1	2	2267	CCDC25	3
752	IFI6	1	1508	TUBB4B	2	2268	ARHGEF2	3
753	ZNF766	1	1509	IDS	2	2269	AC018653.3	3
754	AC112907.3	1	1510	RORA	2	2270	RUFY2	3
755	MRPL17	1	1511	MAP1LC3B	2	2271	STOML2	3
756	AUH	1	1512	PNRC1	2	2272	CLK2	3
			1513	ERN1	2	2273	ILKAP	3
			1514	RNF139	2	2274	SMCHD1	3
			1515	SMAD7	2			
			1516	DENND4A	2			

Supplementary File 1c

Pro-inflammatory signature	Immune regulatory signature	Interferon responsed signature	Lipid metabolism signature
CCL3	CD274	ISG20	ACP5
CCL4	CD40	ISG15	LPL
CCL20	CD80	IFI44L	TREM2
CCL3L1	CD86	GBP1	CCL18
CCL4L2	IDO1	CASP1	CTSB
CXCL1	ICOSLG	CASP4	CTSD
CXCL2	IL10	CXCL9	CTSL
CXCL3	TGFB1	CXCL10	FABP5
CXCL5		CXCL11	FABP4
CXCL8		IFIT1	ALOX5 AP
IL1B		IFIT2	
AREG		IFIT3	
EREG		IFITM1	
HBEGF		IFITM3	

Neutrophil maturation	Neutrophil chemotaxis	Phagocytosis	Type I interferon signaling pathway	Chemokine activity
SELPLG	BSG	A0A087WW49	STAT2	C5
SAT1	C1QBP	MYH9	IFNAR1	CCL1
GRINA	C3AR1	IGHV4-59	CDC37	CCL11
CCL23	C5AR1	IGHV4-39	H7C3V1	CCL13
CCL15	C5AR2	IGHV2-5	PTPN6	CCL14
CCL14	CAMK1D	IGHV3OR16-9	IFNA8	CCL15
CEBPB	CCL1	TREM2	IFIT5	CCL16
ANXA2	CCL11	IGHV2-70	TBK1	CCL17
GDA	CCL13	IGKC	GBP2	CCL18
CLEC4D	CCL14	TRBC1	PTPN2	CCL19
CLEC4E	CCL15	IGHG1	MX2	CCL2
MMP9	CCL16	IGHM	MX1	CCL20
TMCC1	CCL17	IGHA1	SAMHD1	CCL21
AC068580.4	CCL18	IGHV1-69-2	ISG20	CCL22
CTSD	CCL19	RAB31	HLA-F	CCL23
ARG2	CCL2	TRBC2	IP6K2	CCL24
FPR1	CCL20	CDC42	YTHDF2	CCL25
SLC16A3	CCL21	THBS1	ZBP1	CCL26
JUNB	CCL22	IGLL5	LSM14A	CCL27
DUSP1	CCL23	IGLC2	CNOT7	CCL28
RDH12	CCL24	IGHV4-30-4	IRF3	CCL3
SLC7A11	CCL25	IGHV3-43D	TYK2	CCL3L1
ASPRV1	CCL26	IGHV3-30-5	IKBKE	CCL4
S100A11	CCL3	IGHV3-30-3	HLA-G	CCL4L1
TIMP2	CCL3L1	IGHV1-8	STAT1	CCL5
MXD1	CCL4	ARHGAP25	IRF1	CCL7
CYP4F3	CCL4L1	IGLL1	HLA-C	CCL8
MAP1LC3B2	CCL5	GULP1	PTPN11	CKLF
MAP1LC3B	CCL7	TRDC	ISG15	CX3CL1
YPEL3	CCL8	RHOG	USP18	CXCL1
CCR1	CCR7	PPARG	TREX1	CXCL10
FTL	CD300H	IGHV4-31	IRF8	CXCL11
IL36G	CD74	IGHV4-38-2	IRF6	CXCL12
SLPI	CKLF	IGLC3	IFIT3	CXCL13
RETNLB	CSF3R	ABCA1	IRF9	CXCL14
CSTA	CX3CL1	STAP1	IFNA16	CXCL16
CD300LF	CXADR	XKR6	IFNA4	CXCL2
FTH1	CXCL1	XKR7	IFNA6	CXCL3
HACD4	CXCL10	XKR9	TTLL12	CXCL5
MSRB1	CXCL11	IGHV2-70D	HLA-E	CXCL6
IFITM1	CXCL13	IGHV3-66	HLA-H	CXCL8
IFITM2	CXCL2	IGHV4-61	HLA-B	CXCL9
IFITM3	CXCL3	IGHV1-58	IFI27	GPR15L
MMP8	CXCL5	IGHV5-51	IRF2	PF4
S100A6	CXCL6	IGHV3-38	PTPN1	PF4V1
CXCR2	CXCL8	IGHV3-35	C9JQL5	PPBP
IL1B	CXCL9	IGHV4-28	JAK1	XCL1
STK17B	CXCR1	IGHV1-24	IRF5	XCL2
	CXCR2	IGHV3-20	ADAR	
	DAPK2	IGHV1-18	BST2	

DNM1L	IGHV3-16	RNASEL
DPEP1	IGHV1-3	EGR1
DPP4	ITGA2	IFNA14
EDN1	MARCO	IFNA7
EDN2	CD300A	IFNA1
EDN3	VAMP7	IFI35
FCER1G	S4R3C0	HSP90AB1
GBF1	HAVCR1	MAVS
ITGA1	RAC1	MYD88
ITGB2	ARHGAP12	IRF4
JAML	BIN2	CACTIN
LBP	APPL2	IRF7
LGALS3	MFGE8	RSAD2
MCU	MEGF10	MMP12
MDK	FCGR1A	OASL
MOSPD2	IGLC7	IFNAR2
NCKAP1L	XKR5	PSMB8
PDE4B	XKR4	SP100
PF4	A0A0J9YWU9	H0Y3Z8
PF4V1	GSN	ABCE1
PIK3CD	IGHV4-4	HLA-A
PIK3CG	IGHV1-2	NLRC5
PIKFYVE	C3	WNT5A
PIP5K1C	ITGAM	METTL3
PLA2G1B	IGHV5-10-1	IFIT1
PPBP	A0A0J9YY99	IFIT2
PPIA	ABCA7	IFI6
PPIB	SH3BP1	IRAK1
PREX1	MSR1	IFNA2
RAC1	IGLC1	IFNA10
RAC2	ALOX15	IFNA21
RIPOR2	ELMO1	IFNA5
S100A12	IGHV1-69	IFNA17
S100A8	NCKAP1L	IFNB1
S100A9	RHOBTB2	IFITM1
SAA1	IGHV1-69D	UBE2K
SLIT2	IGHV4-34	FADD
SRP54	RHOBTB1	TRIM6
SYK	IGHV3-64D	OAS2
TGFB2	IGLC6	XAF1
THBS4	IGHV3-9	MUL1
TIRAP	IGHV3-7	DCST1
URS00000B7E30_9606	IGHV3-33	IFITM2
VAV1	IGHV3-30	IFITM3
VAV3	IGHV3-53	YTHDF3
XCL1	IGHV3-13	OAS1
XCL2	IGHV3-23	OAS3
	IGHV3-48	
	IGHV3-11	
	IGHV1-46	
	IGHV6-1	
	IGHV3-21	
	IGHV2-26	
	IGHV3-73	
	IGHV7-4-1	
	A0A0J9YW62	
	ITGB2	
	FCER1G	
	IGHV1OR21-1	
	GATA2	
	ADGRB1	
	IGHV3-15	
	IGHV7-81	
	DOCK1	
	XKR8	
	IGHV3-43	
	IGHD	
	IGHA2	

		IGHG4
		IGHG3
		IGHG2
		IGHE
		RAC2
		IGHV1OR15-9
		IGHV2OR16-5
		IGHV3-72
		IGHV3-74
		IGHV4-30-2
		IGHV3-49
		IGHV1-45
		FCGR2B
		IGHV3OR16-8
		IGHV3OR16-10
		IGHV3OR16-13
		IGHV3OR15-7
		IGHV1OR15-1
		IGHV3OR16-12
		IGHV4OR15-8
		ANO6
		F2RL1
		AIF1
		RHOH
		CD36
		IGHV3-64

**Exhaustion**	**Cytotoxicity**	**Treg**
TIGIT	KLRG1	IL2RA
PDCD1	KLRD1	FOXP3
CTLA4	GZMK	
CD160	GZMB	
KLRC1	GZMA	
BTLA	GNLY	
HAVCR2	PRF1	
LAG3	GZMM	
	NKG7	
	TBX21	
	ZEB2	
	HOPX	
	GZMH	
	KLRK1	
	IFNG	
	CCL3	
	CST7	
	ADGRG1	
	IL32	
	CRTAM	
	CX3CR1	
	KLRC1	
	FGFBP2	
	FCGR3A	
	NCR3	
	CCL4	

Supplementary File 1d

Celltype	Abbreviation	Markers	Reference
Hepatocytes	Hepatocytes	ALB,TTR,APOA1	10.1186/s13059-020-02210-0,10.1016/j.cell.2020.11.041,10.1016/j.cell.2020.11.041
Cholangiocytes	Cholangiocytes	KRT19,KRT7,CFTR	10.1186/s13059-020-02210-0,10.1186/s13059-020-02210-0,10.1186/s13059-020-02210-0
Endothelial cells	ECs	PECAM1,VWF,CDH5	10.7150/thno.54917,10.1158/1078-0432.CCR-19-3231,10.1038/s41556-019-0446-7
Hepatic stellate cells	HepSCs	PDGFRB,ACTA2,RGS5	10.1172/JCI146987,10.1016/j.cell.2020.11.041,10.1016/j.cell.2020.11.041
Proliferating cells	ProliferatingCells	MKI67,TOP2A,STMN1	10.1158/1078-0432.CCR-19-3231,10.1681/ASN.2019080832,10.1038/s41467-019-14256-1
B cells	BCells	CD79A,MS4A1,CD19	10.1038/s41467-021-21795-z,10.1038/s41467-021-21795-z,10.1038/s41422-020-0378-6
Plasma cells	PlasmaCells	JCHAIN,CD79A,MZB1	10.1038/s41467-021-21795-z,10.1038/s41467-021-21795-z,10.1038/s41467-019-14256-1
T and NK cells	TandNK	CD3D,CD3E,NKG7	10.1038/s41467-021-21795-z,10.1016/j.immuni.2019.09.008,10.1530/ERC-22-0325
Neutrophils	Neutrophils	FCGR3B,S100A9,S100A8	10.1038/s41467-021-22801-0,10.1038/s41467-021-22801-0,10.1038/s41467-021-22801-0
Mast cells	MastCells	TPSAB1,TPSB2,CPA3	10.1158/1078-0432.CCR-19-3231,10.1038/s41467-021-22801-0,10.1016/j.immuni.2019.09.008
Mononuclear phagocytes	MPs	CD14,CSF1R,HLA-DRA	10.1038/s41586-019-1631-3,10.1038/s41586-019-1631-3,10.1038/s41586-019-1631-3
Plasmacytoid dendritic cells	pDCs	IL3RA,CLEC4C,LILRA4	10.1158/1078-0432.CCR-19-3231,10.1158/1078-0432.CCR-19-3231,10.1038/s41593-020-00789-y

Celltype	Abbreviation	Markers	Reference
Proliferating cells	ProliferatingCells	MKI67,TOP2A,STMN1	10.1158/1078-0432.CCR-19-3231,10.1681/ASN.2019080832,10.1038/s41467-019-14256-1
Macrophages	Macrophages	CD14,C1QA,CSF1R	10.1158/1078-0432.CCR-19-3231,10.1016/j.immuni.2019.09.008,10.1038/s41556-019-0446-7
Monocytes	Monocytes	LYZ,FCN1,VCAN	10.1038/s41467-019-11049-4,10.1038/s41467-021-22801-0,10.1038/s41467-021-22801-0
Mature dendritic cells	MatureDCs	LAMP3,CCR7,CD83	10.1158/2159-8290.CD-19-0138,10.1038/s41591-021-01323-8,10.1158/2159-8290.CD-19-0138
Conventional type 1 dendritic cells	cDC1	CLEC9A,XCR1,IRF8	10.1038/s41586-019-1652-y,10.1038/s41586-019-1652-y,10.1038/s41422-020-0374-x
Conventional type 2 dendritic cells	cDC2	CD1C,CLEC10A,FCER1A	10.1038/s41586-019-1652-y,10.1038/s41586-019-1652-y,10.1038/s41593-020-00789-y
Kupffer cells	KCs	MARCO,CD5L,VCAM1	10.1016/j.cell.2020.11.041,10.1016/j.cell.2020.10.048,10.1038/s41586-019-1631-3

Celltype	Abbreviation	Markers	Reference
Group 3 innate lymphoid cells	ILC3	IL1R1,IL23R,KIT	10.1038/s41590-019-0425-y,10.1038/s41590-019-0425-y,10.1038/s41586-021-03852-1
NK T cells	NKT	NKG7,CD3D,GNLY	10.7150/thno.48201,10.7150/thno.48201,10.7150/thno.48201
Natural killer cells	NK	NKG7,GNLY,NCAM1	10.1038/s41392-020-00248-x,10.1038/s41467-021-21795-z,10.1126/science.aad0501
CD4+ naive T cells	CD4NaiveT	CCR7,SELL,LEF1	10.1038/s41467-021-22164-
CD4+ memory T cells	CD4Tmem	CD4,IL7R,CD40LG	10.1182/blood-2018-08-862292,10.1016/j.immuni.2020.12.011,10.1038/s41467-022-33170-7
CD4+ regulatory T cells	CD4Treg	CTLA4,FOXP3,IL2RA	10.1038/s41421-020-0157-z,10.1038/s41421-020-0157-z,10.1038/s41421-020-0157-z
CD8+ mucosal-associated invariant T cells	CD8MAIT	KLRB1,SLC4A10,NCR3	10.1016/j.cell.2021.05.013,10.1186/s13059-019-1906-x,10.1038/s41392-020-00263-y
CD8+ effector T cells	CD8Teff	CD8A,GZMK,CD3D	10.1038/s41467-021-22801-0,10.1038/s41591-019-0590-4,10.1016/j.cell.2020.06.001
